# Genomic insights into evolution and control of *Wohlfahrtia magnifica*, a widely distributed myiasis‐causing fly of warm‐blooded vertebrates

**DOI:** 10.1111/1755-0998.13654

**Published:** 2022-06-18

**Authors:** Zhipeng Jia, Surong Hasi, Claus Vogl, Pamela A. Burger

**Affiliations:** ^1^ Department of Interdisciplinary Life Sciences, Research Institute of Wildlife Ecology University of Veterinary Medicine Vienna Vienna Austria; ^2^ Key Laboratory of Clinical Diagnosis and Treatment Technology in Animal Disease Hohhot China; ^3^ Department of Biomedical Sciences, Institute of Animal Breeding and Genetics University of Veterinary Medicine Vienna Vienna Austria

**Keywords:** comparative genomics, de novo assembly, fly species, gene family, low DNA input, PacBio HiFi sequencing, positively selected genes

## Abstract

*Wohlfahrtia magnifica* is a pest fly species, invading livestock in many European, African and Asian countries, and causing heavy agroeconomic losses. In the life cycle of this obligatory parasite, adult flies infect the host by depositing the first‐stage larvae into body cavities or open wounds. The feeding larvae cause severe (skin) tissue damage and potentially fatal infections if untreated. Despite serious health detriments and agroeconomic concerns, genomic resources for understanding the biology of *W. magnifica* have so far been lacking. Here, we present a complete genome assembly from a single adult female *W. magnifica* using a Low‐DNA Input workflow for PacBio HiFi library preparation. The de novo assembled genome is 753.99 Mb in length, with a scaffold N50 of 5.00 Mb, consisting of 16,718 predicted protein‐encoding genes. Comparative genomic analysis revealed that *W. magnifica* has the closest phylogenetic relationship to *Sarcophaga bullata* followed by *Lucilia cuprina*. Evolutionary analysis of gene families showed expansions of 173 gene families in *W. magnifica* that were enriched for gene ontology (GO) categories related to immunity, insecticide‐resistance mechanisms, heat stress response and cuticle development. In addition, 45 positively selected genes displaying various functions were identified. This new genomic resource contributes to the evolutionary and comparative analysis of dipterous flies and an in‐depth understanding of many aspects of *W. magnifica* biology. Furthermore, it will facilitate the development of novel tools for controlling *W. magnifica* infection in livestock.

## 1 INTRODUCTION

The obligate parasitic spotted flesh fly, *Wohlfahrtia magnifica* (Diptera: Sarcophagidae), is globally distributed, ranging from North Africa, through eastern and southwestern Europe extending to northeast Asia (Farkas et al., 1997; Gaglio et al., 2011; Giangaspero et al., 2011; Hall et al., 2009; Ruiz Martinez & Leclercq, 1994; Sotiraki et al., 2010; Valentin et al., 1997; Yasuda, 1940). It is a major myiasis‐causing fly and can infect live mammals (Schnur et al., 2009), especially livestock, such as horses (Farkas & Képes, 2001; Yan et al., 2019), sheep (Dehghani et al., 2014; Farkas et al., 1996), camels (Moshaverinia et al., 2013; Valentin et al., 1997), and even occasional reports of infected humans (ÇiftÇio et al., 1996; Kokcam & Saki, 2005). Similar to other dipterous larvae in the family Calliphoridae that infect livestock, e. g., *Lucilia cuprina* (Anstead et al., 2015) and *Cochliomyia hominivorax* (Scott et al., 2020), adult *W. magnifica* females seek their hosts and lay their larvae on the predisposed skin of the genitalia or open wounds. The larvae feed on the host's tissues for development. As a result, the numerous bites of the larvae enlarge the wound and lead to severe tissue damage within only a few days. This may cause emaciation, reduction of productivity, reproductive disorders, and if untreated, heavy infections possibly leading to death (Farkas et al., 1997; İpek et al., 2012; Martinez et al., 1987).

Economic loss owing to *W. magnifica* infection in livestock worldwide is considerable (Hall, 1997; Ruiz‐Martínez et al., 1987). In China, field surveys show that approximately 20% of female Bactrian camels are infected each year. In Europe, the individual prevalence of *W. magnifica* ranges from 0.7% to 95%, especially in Spain and Italy (Hall & Farkas, 2000; Sotiraki et al., 2012). For example, Remesar et al. (2022) surveyed 122 flocks of 73,683 sheep in Albacete Province of Spain, and the result showed that 90% of flocks were infected and the prevalence of the individuals was 7.1%. Over many years, different control methods have been employed, mainly applying insecticides to control larval development (Farkas et al., 1996; Giangaspero et al., 2011; Sotiraki et al., 2005). However, insecticides cannot prevent the infection/reinfection and may cause undesired side effects, for example, local corrosion and necrosis of skin and tissue at the site of infection, leading to festering wounds, prone to secondary infection (Hall & Farkas, 2000). In addition, the excessive use of insecticides could cause the emergence of insecticide resistance. Therefore, the existing approaches for the control of infection with the *W. magnifica* are still limited and not applicable for long‐term use. New alternative control tools, such as vaccine‐based approaches, sterile insect technique (SIT), development and discovery of new drugs, or other genetic, immunological or chemical control strategies are greatly needed.

Until today, most research on *W. magnifica* has focused on epidemiological studies (Ruiz Martínez et al., 1993), morphological observations (Li et al., 2020; Szpila et al., 2014; Yasuda, 1940), and investigation of life history (Cruz et al., 1996), while little is known about the host's immune response, parasite–host interaction, or regulatory mechanisms on the level of molecular biology. A high‐quality genome is a fundamental resource for understanding many aspects of the developmental and reproductive biology, physiology and biochemistry as well as complex pathogenic mechanisms of *W. magnifica* or for developing novel control methods preventing fly invasion to livestock. However, due to the presence of insect‐highlighting features such as high polymorphism, high‐quality genome assembly is difficult to obtain (Richards & Murali, 2015). A representative obstacle is that repetitive sequences or polymorphic regions cannot be straddled well, leading to a fragmented genome assembly with lower contig N50 lengths. As long‐read technologies have the inherent advantages of spanning polymorphic regions, repetitive sequences and transposable elements (TE) (Richards & Murali, 2015), at present, more and more insect genome projects are a combination of continuous long reads from PacBio/Nanopore and short reads from Illumina (Meng et al., 2020; Ren et al., 2021; Ye et al., 2021). Using the strategy, to date, a number of full and draft genomes of agricultural pests are completed and publicly available, such as Mediterranean fruit fly (*Ceratitis capitata*) (Papanicolaou et al., 2016), sheep blow fly (*L. cuprina*) (Anstead et al., 2015), and the New World screwworm fly (*C. hominivorax*) (Scott et al., 2020). This strategy requires a sufficiently high quantity of DNA for library preparation for insects with small physical sizes, which can normally be obtained from the time‐consuming rearing of inbred lines. However, some attributes of *W. magnifica* have severely hindered a high‐quality genome assembly so far as insufficient DNA quantities obtained from a single physically small adult fly have posed a major problem. One way to overcome this challenge would be to pool inbred individuals to obtain sufficient DNA for library preparation. While this method works well for some organisms that can be inbred, such as *Drosophila melanogaster* (Adams et al., 2000), unfortunately, *W. magnifica* is notoriously difficult to rear (in vitro rearing) (Farkas et al., 2005). In the laboratory, *W. magnifica* has a very high mortality rate, which renders the inbreeding strategy unfeasible. For example, researchers used dead animals or their tissues as larval diets to rear *W. magnifica*, unfortunately without success (Ruiz Martinez et al., 1992; Soler Cruz et al., 1998). Another attempt would be to feed on the artificial diet and the results showed 64%–98% mortality in the larval stage, 61%–100% mortality in the pupal stage, and only a maximum of 6% were successfully reared from the first stage larvae to the adult stage (Farkas et al., 2005).

The availability of the high fidelity (HiFi) library preparation workflow from low‐DNA input (Kingan et al., 2019) has improved this situation. Compared to the standard HiFi library preparation of PacBio, which requires relatively large DNA amounts (a minimum input of 5 μg high‐molecular‐weight genomic DNA is recommended for the Sequel II systems), this workflow significantly reduces DNA requirement. As a result, very small amounts of genomic DNA (>400 ng for the Sequel II system) isolated from a single insect can produce sufficient amounts of sequencing data for a high‐quality genome assembly of up to 1 Gb using only one SMRT cell combined with the circular consensus sequencing (CCS) mode of the PacBio Sequel II system. This also avoids time‐consuming inbreeding and pooling requirements.

Here, we report a high‐quality and accurate genome assembly of *W. magnifica* with the size of 753.99 Mb, including complete annotation. This resource can assist in enlightening the genetic mechanisms of *W. magnifica* and eventually in developing applications to control this invasive fly species. Finally, we performed comparative genome analyses with other dipterous flies, allowing us to gain a better understanding of the molecular evolution of *W. magnifica*.

## 2 MATERIALS AND METHODS

### 2.1 Sample collection

The study site is located at the Camel Culture Base in Siziwang Banner, Ulanqab City (Inner Mongolia Autonomous Region, China). The sample collection was performed in the frame of veterinary health monitoring and treatment of Bactrian camels with *W. magnifica* infection. The first, second, and third stages of *W. magnifica* larvae were obtained from the genitalia of an infected female Bactrian camels in a lie‐down position, of which the first and second stages were used for RNA extraction (Z. Jia, S. Hasi, C. Vogl, P. A. Burger, unpublished data). For adult fly rearing, the third‐stage larvae were placed in preprepared foam boxes with the local soil. After the collected larvae had burrowed into the soil, the foam boxes were brought to the laboratory and placed in a dry place for hatching. Around 18–20 days, when all the third‐stage larvae had emerged as adult flies, the adult females with the largest relative body size were selected and frozen at −80°C until DNA and RNA extraction.

### 2.2 DNA isolation and sequencing

High‐molecular‐weight genomic DNA was extracted from a single adult female *W. magnifica*. The quantity of extracted DNA was measured using an Invitrogen Qubit 3.0 fluorometer (Thermo Fisher Scientific) and Nanodrop NC2000 (Thermo Fisher Scientific), and the integrity of extracted DNA was estimated on a 1.2% agarose gel to check for any degradation. With approximately 1.215 μg DNA isolated from a single female *W. magnifica* (Table S1), a 10 kb HiFi library was prepared following the procedure and recommendations of the kit: Preparing HiFi Libraries from low DNA input using SMRTbell Express Template Prep Kit 2.0 (Pacific Biosciences). In short, genomic DNA was sheared to average size distribution of 10 kb using g‐TUBEs (Covaris) and subsequently purified. Purified DNA fragments were added to the enzyme reaction tubes and incubated at 37°C for 15 min to remove single‐strand overhangs followed by the addition of repair mix and incubation at 37°C for 30 min to repair the damage within the DNA backbone. After DNA damage repair, the ends of the double‐stranded fragments were polished and subsequently tailed with an A‐overhang by adding End Prep Mix and incubating at 20°C for 10 min and then at 65°C for 30 min. Ligation with T‐overhang SMRTbell adapters occurred at 20°C for 60 min, after which the AMPure PB beads (Pacific Biosciences) were employed to purify the SMRTbell library. Due to the presence of short fragments after the first purification step, the library was size‐selected with AMPure PB beads (Pacific Biosciences) to remove SMRTbell fragments less than 3 kb. Subsequently, the size distribution and quantity of the SMRTbell library to be sequenced were measured using Invitrogen Qubit 3.0 Fluorometer (Thermo Fisher Scientific) and Agilent 2100 Bioanalyser (Agilent Technologies). The final SMRTbell library was sequenced on the Pacbio Sequel II system with a single SMRT Cell 8M.

### 2.3 RNA extraction, library preparation, sequencing and data filtering

To assist the assessment of genome assembly and genome annotation, we sequenced transcriptome data from the first‐ and second‐stage larvae and adult flies of *W. magnifica* with three replicates for each sample. Total RNA was extracted from each sample. RNA quality was examined by agarose gel electrophoresis and Agilent 2100 Bioanalyser (Agilent Technologies). Library preparation followed the instructions of TruSeq Stranded mRNA LT Sample Prep Kit (Illumina). Briefly, mRNA was enriched by binding to poly‐A on mRNA with Beads containing oligo‐dT, and the enriched mRNA was interrupted to a 200–300 bp fragment. Then, the fragmented RNA was used as a template for reverse transcription to the first‐strand complementary DNA (cDNA) synthesis, followed by the synthesis of second‐strand cDNA, which uses the first‐strand cDNA as a template. After that, synthetic double‐stranded cDNA was end‐repaired, poly (A) added, and ligated to Illumina sequencing adapters. The ligation products were first purified by removing the free adaptor and the fragment without the attached adaptor, and next amplified by PCR using specific primers. Finally, the prepared libraries were sequenced on an Illumina NovaSeq platform. To obtain high‐quality clean reads, the raw paired‐end reads were trimmed by removing adapter sequences and low‐quality reads using bbmap (https://sourceforge.net/projects/bbmap/).

### 2.4 Genome assembly

To correct sequencing errors and generate highly accurate consensus reads, we converted raw reads into circular consensus sequences (CCS; hereafter HiFi sequences) using the program ccs version 5.0.0 with default settings (https://github.com/PacificBiosciences/ccs). Next, we used icecreamfinder version 38.84 (https://sourceforge.net/projects/bbmap/) to filter out and/ or trim HiFi sequences with inverted repeats and remaining adapter sequences with default settings. Then, we filtered the resulting HiFi reads for potential bacterial contamination using sendsketch version 38.87 (https://sourceforge.net/projects/bbmap/) to send a reduced representation of the trimmed/ filtered HiFi reads against drafts from the NCBI nucleotide database inspecting up to 1000 records in the results. We used NCBI data sets version 10.9.0 to retrieve matching bacterial genome sequences and seal version 38.87 (https://sourceforge.net/projects/bbmap/) with k = 31 and minkmerfraction = 0.5 to assign and remove HiFi sequences with at least 50% of each HiFi sequence's 31‐mers matching the bacterial genomes. For the genome assembly based on the filtered HiFi sequences, the official PacBio software for HiFi genome assembly, the improved phased assembler (IPA) version 1.3.2 (https://github.com/PacificBiosciences/pbbioconda/wiki/Improved‐Phased‐Assembler), was employed with default settings.

To assess the completeness of the genome assembly, we applied the genome mode of the Benchmarking Universal Single‐Copy Orthologues (busco, version 4.0.6) (Simão et al., 2015) and searched for conserved single‐copy genes belonging to the core gene sets of diptera_odb10 (Creation date: 2020‐08‐05, number of species: 56, number of BUSCOs: 3285). In addition, we compared the BUSCO scores between the *W. magnifica* genome assembly and other dipterous flies, including *Lucilia cuprina* (ASM118794v1), *Musca domestica* (MdomA1), *Stomoxys calcitrans* (ScalU1), *Glossina morsitans* (GmorY1), *Drosophila melanogaster* (BDGP6.32), *Mayetiola destructor* (Mdes_1.0), *Aedes aegypti* (AaegL5), *Anopheles gambiae* (AgamP4) from Ensembl Metazoa and *Sarcophaga bullata* (GCA_005959815.1) from NCBI.

### 2.5 Annotation of repetitive sequences

We soft‐masked (converted uppercase to lowercase bases) the genome assembly by generating a species‐specific repeat library with repeatmodeler version 2.0.1(http://www.repeatmasker.org/RepeatModeler/) using ‐engine ncbi and ‐LTRStruct. The repeat library from repeatmodeler was filtered to remove known uniprot/swissprot version 2020_05 proteins using protexcluder version 1.1 (Campbell et al., 2014). We then used repeatmasker version 4.1.1 (http://www.repeatmasker.org/) with the options “‐xsmall ‐a” and with the species‐specific repeat library to identify repetitive sequences.

### 2.6 Gene annotation

We annotated the genome assembly with braker version 2.1.5 (Hoff et al., 2019), Augustus version 3.3.3 (Stanke et al., 2004), and genemarkes version 4.6.3 (Lomsadze et al., 2005). We used proteins from Arthropoda v100_odb10 (Kriventseva et al., 2019), RNA‐Seq alignments made between RNA‐Seq libraries aligned to the genome with hisat2 version 2.2.1 (Kim, Nam, et al., 2019; Kim, Paggi, et al., 2019) using ‐‐max‐intronlen 100,000 and ‐‐dta. For BRAKER we used the softmasking, etpmode, and the following augustus settings: ‐‐alternatives‐from‐sampling = true –minexonintronprob = 0.2 –minmeanexonintronprob = 0.5 –sample = 100 –maxtracks = 3 –temperature = 2. Then, we employed maker version 3.01.03 (Cantarel et al., 2008) to merge the annotations by Augustus and GeneMark using the hintsfile.gff produced by braker as the protein_gff passed to maker and the concatenated augustus.hints.gtf and GeneMark‐ETP's genemark.f.multi_anchored.gtf filtered by gffread version 0.12.3 (Pertea & Pertea, 2020) using the settings ‐‐adj‐stop ‐J ‐‐sort‐alpha ‐E ‐‐keep‐genes as the pred_gff passed to MAKER. We functionally annotated the MAKER filtered genes using proteins with a combination of blastp searches against UniProt/Sprot release 2020_05 implemented with diamond version 2.0.4 (Buchfink et al., 2015) using the settings ultra‐sensitive, evalue 1e‐6 and max‐target‐seqs 1. The resulting annotations were reformatted with GAG (http://genomeannotation.github.io/GAG/; Geib et al., 2018) and Annie (http://genomeannotation.github.io/annie/).

In addition, we annotated the noncoding RNA genes, including transfer RNA (tRNA) genes and ribosomal RNA (rRNA) genes, within the genome assembly. trnascan‐se version 2.0 (Lowe & Eddy, 1997) with Eukaryotic parameters were used to predict the tRNA genes. The rRNA genes were annotated using rnammer version 1.2 (Lagesen et al., 2007) with default parameters.

### 2.7 Phylogenetic analysis

To determine the phylogenetic relationship among the dipterous flies, we retrieved protein sets of *Lucilia cuprina* (ASM118794v1), *Musca domestica* (MdomA1), *Stomoxys calcitrans* (ScalU1), *Glossina morsitans* (GmorY1), *Drosophila melanogaster* (BDGP6.32), *Mayetiola destructor* (Mdes_1.0), *Aedes aegypti* (AaegL5), *Anopheles gambiae* (AgamP4) from Ensembl Metazoa and *Sarcophaga bullata* (GCA_005959815.1) from NCBI. For genes with more than two transcripts within genomes, we only kept the protein sequence of the longest transcript. Then, gene families were clustered with orthofinder version 2.5.1 with the settings: ‐M msa ‐S blast ‐A mafft ‐T fasttree (Emms & Kelly, 2015, 2019), which specifies multiple sequence alignments (−M) for the gene tree inference. The protein sequences of the resulting single‐copy genes were aligned using mafft version 7.475 (Katoh & Standley, 2013) with default parameters, followed by trimming with gBlocks to remove gaps (Talavera & Castresana, 2007). After trimming, we used seqkit version 0.10.0 (Shen et al., 2016) to concatenate the trimmed protein sequences of single‐copy orthologous of each species into one super gene. We used prottest version 3.4.2 (Darriba et al., 2011) to determine the optimal amino acid substitution model. Subsequently, the phylogenetic trees were inferred using raxml version 8.2.12 (Stamatakis, 2014) using the PROTGAMMALG substitution model with 1000 bootstrap replicates.

To estimate divergence times among species, the MCMCTree program of paml version 4.9 (Yang, 2007) was employed and five calibration points were obtained from the TimeTree database (http://www.timetree.org/), including *M. domestica*–*S. calcitrans* (27–37 million years ago [Ma]), *M. domestica*–*L. cuprina* (47–71 Ma), *M. domestica*–*G. morsitans* (48–74 Ma), *M. domestica*–*D. melanogaster* (107–172 Ma), and *A. gambiae*–*A. aegypti* (52–147 Ma).

### 2.8 Analysis of parasitism‐related genes

To identify genes associated with parasitism, we selected the protein sets with the longest transcript of three myiasis‐causing flies, including *C. hominivorax*, *L. cuprina* and *W. magnifica*, and *D. melanogaster*, which feeds on rotting fruit, and clustered their gene families using OrthoFinder version 2.5.1 with the same settings as above. Then, the genes in the resulting gene families shared by three myiasis‐causing flies and absent in *D. melanogaster* were further annotated and analysed.

### 2.9 Gene family expansion and contraction

Based on the clustering of gene families generated by OrthoFinder and the phylogenetic relationship with divergence times determined by RAxML and MCMCTree, we used café version 4.1 (De Bie et al., 2006) to analyse the expansion and contraction of gene families, which uses a birth and death process to model gene gain and loss over phylogenetic distance. The resulting expanded genes were extracted for gene ontology (GO) enrichment analysis in OmicShare tools (https://www.omicshare.com/tools) using a false discovery rate (FDR) < 0.05 for multiple test correction.

### 2.10 Positive selection analysis

Including all single‐copy orthologues of the 10 dipterous flies previously inferred by OrthoFinder and their corresponding coding sequences (CDS), we used paraat version 1.0 (Zhang et al., 2012) to align single‐copy orthologues and then back‐translate the multiple protein sequence alignment into a codon alignment with the settings: ‐m mafft ‐g ‐t. Next, the codeml program in the paml package version 4.9 (Yang, 2007) was implemented with the alignment results as inputs using the branch‐site model with the *W. magnifica* branch as foreground and the remaining dipterous fly branches as background. Then, we compared the alternative model (model = 2, NSsites = 2, fix_omega = 0) to the null model (model = 2, NSsites = 2, fix_omega = 1 and omega = 1) using a likelihood ratio test (LRT) calculated with a Chi‐square distribution (*p* < .05; one degree of freedom [*df* = 1]). We corrected for multiple testing using FDR <0.05 and retained only genes that contained amino‐acid sites of positive selection ≥1 as final positive selection candidates.

## 3 RESULTS

### 3.1 Genome assembly and assessment of *W. magnifica*

Since *W. magnifica* cannot be reared in the laboratory, inbred lines cannot be obtained. Taking advantage of the protocol previously described in the method section, which requires a minimum amount of DNA of only >400 ng, we successfully prepared 10 kb Pacbio libraries for sequencing on Pacbio Sequel II System using 1.215 μg of high‐quality genomic DNA extracted from a single female adult *W. magnifica*.

A total of approximately 408 Gb of raw data composed of 43,228,999 reads with 9447 bp average sequence length were produced on a single SMRT Cell 8 M. After converting, a total of approximately 23 Gb of HiFi sequences comprised of 2,197,069 HiFi reads with 10,681 bp average length were obtained, which is approximately 30x coverage based on the genome assembly of *W. magnifica* (Table 1).

**TABLE 1:** Sequencing data statistics of *Wohlfahrtia magnifica*

Parameter	Raw data	Converted HiFi data
Total sequence length (bp)	408,396,865,291	23,467,199,316
Total sequence number	43,228,999	2,197,069
Average sequence length (bp)	9447	10,681
GC content (%)	33.79857849	32.7324404
Max sequence length (bp)	468,940	38,618
N20 (bp)	14,919	15,097
N50 (bp)	10,949	11,580
N90 (bp)	6089	7338

Subsequently, HiFi sequences were assembled using the IPA program. As a result, we obtained a set of 753.99 Mb of primary contigs used as the assembled genome and a set of 647.62 Mb of alternative contigs. The assembled genome has a scaffold N50 of 5.00 Mb, the longest scaffold length of 14.66 Mb, a scaffold number of 543 and a proportion of the bases guanine and cytosine of 32.82% (Table 4). Compared to other dipterous fly genomes, *W. magnifica* has a similar genome size to *M. domestica* but is more than five times larger than the genome of *D. melanogaster*. It is also larger than the genome of *S. bullata*, which belongs to the same family Sarcophagidae, and *C. hominivorax*, which has a similar way of invading livestock to *W. magnifica* (Table 2).

**TABLE 2:** Comparison of summary statistics of genome assembly between *Wohlfahrtia magnifica* and other dipterous flies

Species	*W. magnifica*	*M. domestica*	*D. melanogaster*	*S. bullata*	*C. hominivorax*
Genome size	753.99 Mb	750.40 Mb	143.73 Mb	476.29 Mb	534.08 Mb
Number of scaffolds	543	20,487	1870	42,093	3663
Scaffold N50	5.00 Mb	226.57 kb	25.29 Mb	55.53 kb	616.42 kb
Reference	In the study	GCA_000371365.1	GCA_000001215.4	GCA_005959815.1	Scott et al. (2020) and GCA_004302925.1

**FIGURE 1:**
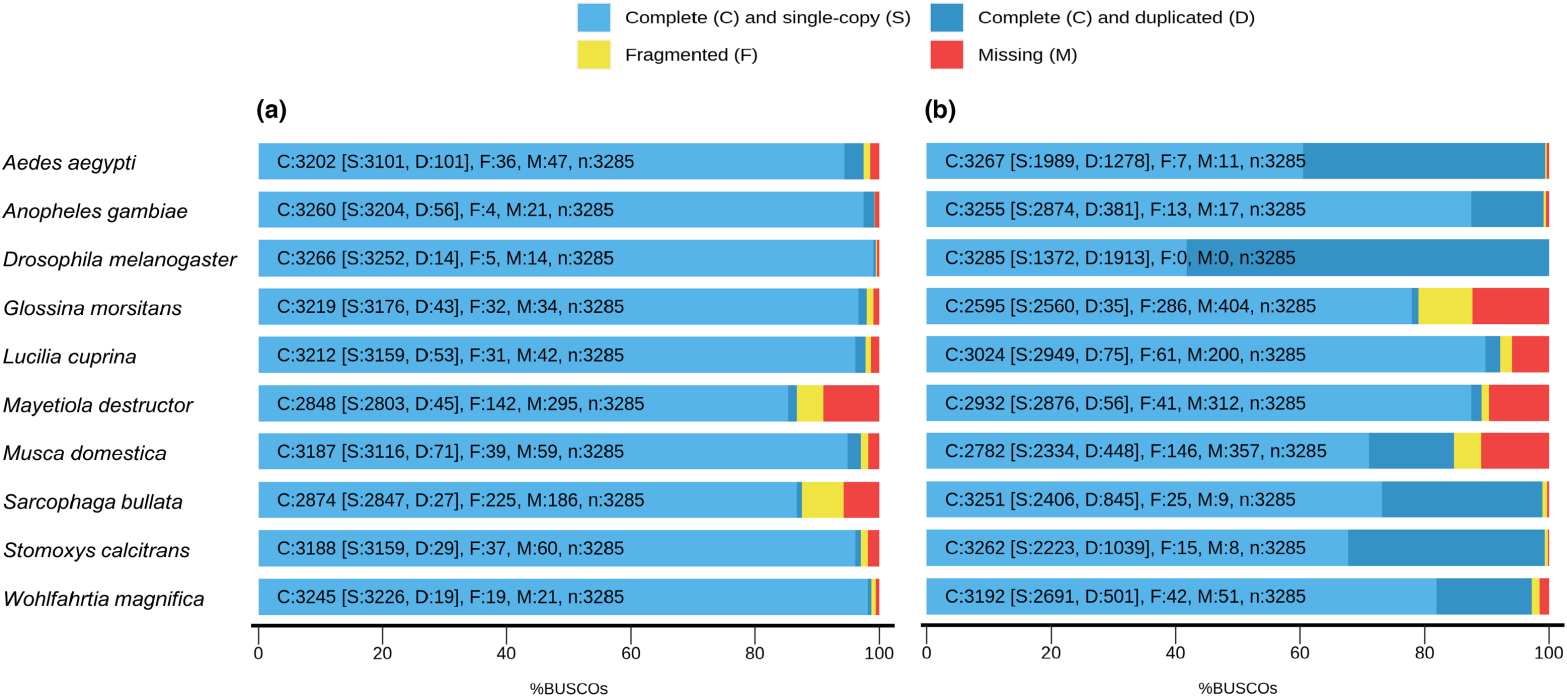
BUSCO analysis between *Wohlfahrtia magnifica* and other nine dipterous flies based on diptera_odb10 gene set. (a) BUSCO assessment results of genomes. (b) BUSCO assessment results of gene sets. C, complete BUSCO genes; S, single‐copy BUSCO genes; D, duplicated BUSCO genes; F, fragmented BUSCO genes; M, missing BUSCO genes; *n*, total number of BUSCO genes

We evaluated the quality of the genome assembly using BUSCO program to search against 3285 conserved single‐copy genes (diptera_odb10). This analysis indicated that 98.8% complete BUSCO genes (3245 genes), including 98.2% complete and single‐copy (3226 genes) and 0.6% complete and duplicated (19 genes), and 0.6% fragmented BUSCO genes (19 genes) could be captured, with only 0.6% missing (21 genes). The BUSCO results were comparable to that of nine publicly available dipterous fly genomes (Figure 1a, Table S2). In addition, we mapped the RNA‐seq data of *W. magnifica* at different developmental larvae stages towards the genome assembly, which was subsequently used in the BRAKER program to aid in the gene structure annotation of the genome, resulting in a mapping rate of 93.62%. Taken together, these results suggested that the genome is complete and accurate.

### 3.2 Annotation of the de novo assembled genome of *W. magnifica*

We searched repeat elements in the assembled genome of *W. magnifica* using Repeatmasker with a species‐specific repeat library generated by RepeatModeler. Overall, identified repetitive sequences accounted for 59.71% (450 Mb) of the total assembled genome, consisting of 56.76% interspersed, 2.48% simple repeat sequences and 0.4% low complexity. Among the interspersed repeats, the most abundant were unassigned sequences (23.37% of the assembled genome), followed by DNA transposons (16.62%), LINEs (12.92%), and LTR elements (3.85%) (Table 3). In addition, we compared the proportion of the genome occupied by repetitive sequences between *W. magnifica* and the other three dipterous flies. The results showed that the repetitive sequences in the genome of *W. magnifica* were similar to *L. cuprina* (57.82%), but significantly higher than those of *C. hominivorax* (45.22%) and *S. bullata* (31.15%) (Table S3).

**TABLE 3:** Repeat element statistics of *Wohlfahrtia magnifica* assembled genome

Repeat element	Numbers	Bases	% of genome
SINEs	0	0	0.00
LINEs	313,495	97,444,031	12.92
LTR	98,752	29,004,031	3.85
DNA	473,914	125,334,368	16.62
Unclassified	976,699	176,211,233	23.37
Simple repeats	279,951	18,690,856	2.48
Low complexity	61,088	2,981,425	0.40
Total	/	450,243,625	59.71

We employed the BRAKER2 pipeline followed by MAKER2 to predict the gene model with the aid of transcriptomic data and protein data (Arthropoda v100_odb10). As a result, 16,718 protein‐encoding genes and 20,017 transcripts were identified in the assembled genome of *W. magnifica*. The longest, shortest gene length and mean gene length as well as mean length for exon, intron and coding sequence are given in Table 4. Of the genes, 64.98% could be functionally annotated in the UniProt/Sprot database. In addition, the gene set was assessed by the BUSCO program with proteins mode, indicating that 97.2% complete conserved single copy genes (diptera_odb10) could be identified, whereas only 1.5% were assigned as missing. This is consistent with other Diptera genomes, suggesting the gene annotation of our de novo assembled genome is of comparable completeness (Figure 1b, Table S2).

**TABLE 4:** Protein‐coding gene annotation statistics of *Wohlfahrtia magnifica* assembled genome

Parameter	
Genome size	753.99 Mb
Number of contigs	543
Contig N50	5.00 Mb
GC content	32.82%
Max scaffold length	14.66 Mb
Number of scaffolds >50 KB	541
Number of genes	16,718
Number of mRNAs	20,017
Number of exons	87,424
Number of introns	67,407
Number of CDS	20,017
Shortest gene	147 bp
Longest gene	394,287 bp
Mean gene length	9789 bp
Mean mRNA length	11,538 bp
Mean exon length	347 bp
Mean intron length	2978 bp
Mean CDS length	1515 bp
Mean exons per mRNA	4
Mean introns per mRNA	3
% of genome covered by genes	21.7%
% of genome covered by CDS	4.0%

Moreover, we identified and annotated noncoding RNA genes in the genome of *W. magnifica*, showing that 576 tRNAs (excluding tRNAs identified as pseudogenes) and 53 rRNAs were identified.

### 3.3 Phylogenetic analysis of *W. magnifica*

**FIGURE 2:**
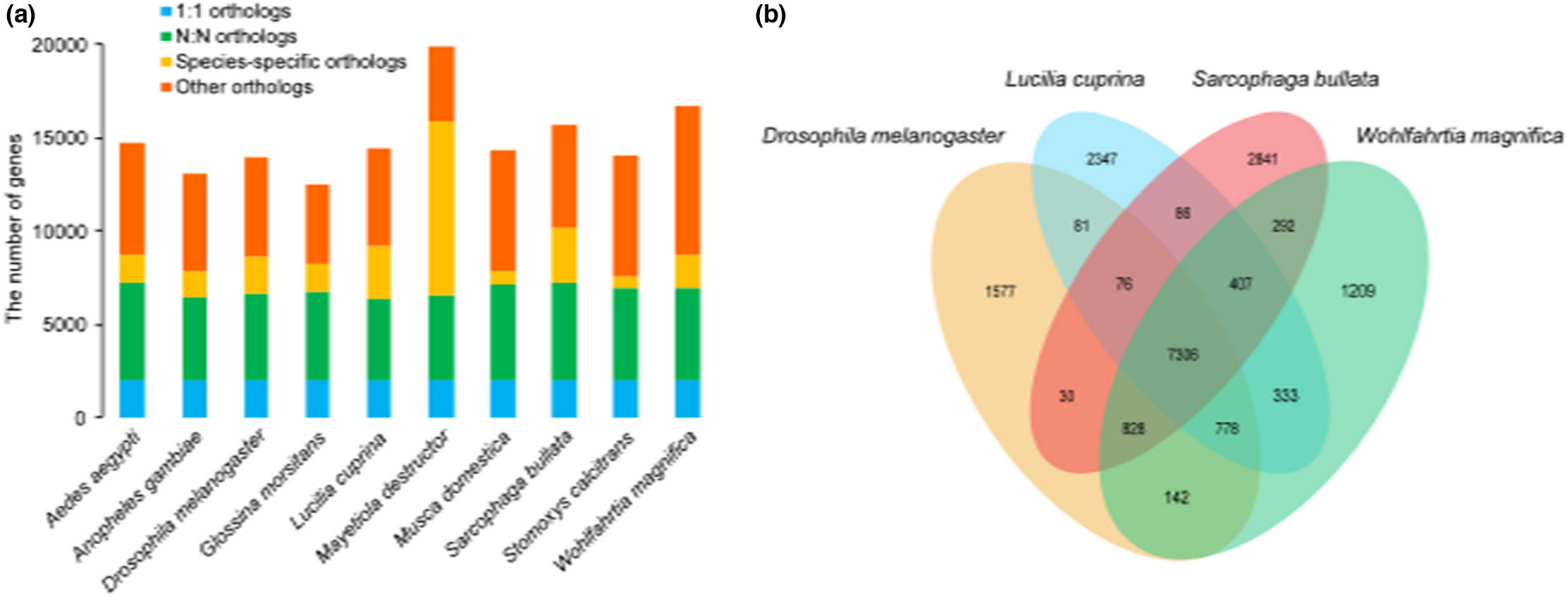
Gene family analysis of *Wohlfahrtia magnifica* and other dipterous flies. (a) Distribution of genes in different species. 1:1 orthologues: Single‐copy orthologues; N:N orthologues: Multiple‐copy orthologues; species‐specific orthologues: Present in specific species; other orthologues: The remaining orthologues. (b) Numbers of orthogroups shared or unique in *W. magnifica*, *S. bullata*, *L. cuprina* and *D. melanogaster*

We searched the orthologues among predicted proteins of *W. magnifica* and those derived from the other nine dipterous flies using OrthoFinder program. A total of 149,614 genes were recovered and 135,230 were grouped into 14,424 orthogroups. The remaining genes were clustered into 14,384 unassigned species‐specific orthogroups, of which each consisted of only one gene. Of 14,424 orthogroups, 2045 orthogroups were found in the gene sets of all 10 flies in single copy form. In addition, we detected 1736 genes present only in the *W. magnifica* genome, including 943 multiple‐copy and 793 single‐copy genes (Figure 2a, Table S4). We also found 7306 orthogroups present in all four dipterous flies, including *W. magnifica* and its closest evolutionary relatives, *S. bullata*, and *L. cuprina*, as well as *D. melanogaster* (Figure 2b).

**FIGURE 3:**
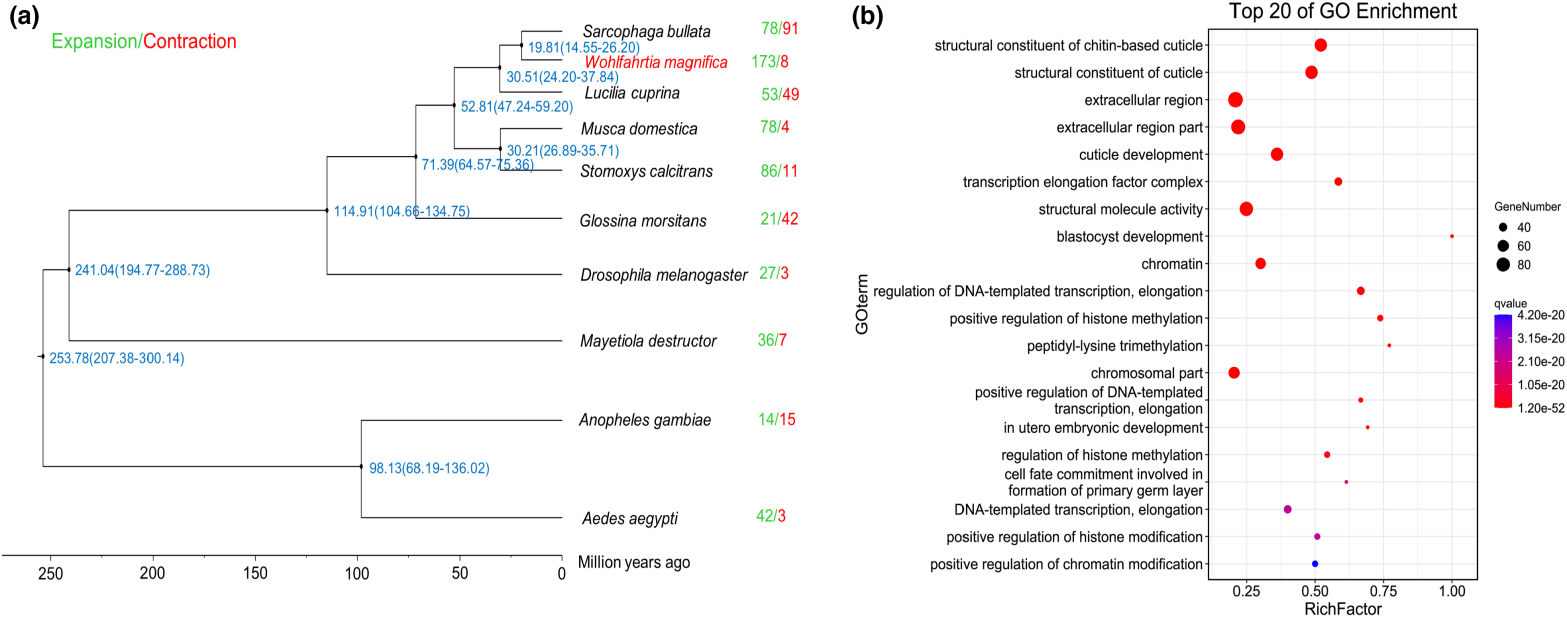
Comparative analysis of the genome of *Wohlfahrtia magnifica*. (a) Phylogenetic relationship, the estimated divergence time (Ma) and gene family of expansion and contraction (the red numbers reflect contracted gene families, whereas the green numbers show expanded gene families.) of *W. magnifica* and other nine dipterous flies. (b) Top 20 of GO enrichment results of 173 expanded gene families of *W. magnifica*

Using the above‐obtained single‐copy orthologues, we performed the phylogenetic reconstruction. Our results suggested that *W. magnifica* is the closest phylogenetic relative to *S. bullata*, as these two dipterous flies belong to the family Sarcophagidae, followed by *L. cuprina*, while being most distant from *A. aegypti* and *A. gambiae* (Figure 3a). As we expected, this result is consistent with other dipteran phylogenetic trees (Martinson et al., 2019; Scott et al., 2020). Furthermore, divergence time estimation revealed that the common ancestors of *W. magnifica* and *S. bullata* split from *L. cuprina* approximately 30.51 Ma, while *W. magnifica* and *S. bullata* divergence time were dated to 19.81 Ma (Figure 3a).

### 3.4 Analysis of parasitism‐related genes

We found 885 gene families shared by three myiasis‐causing flies, but no orthologue in *D. melanogaster*, composed of 1707 genes in *W. magnifica*, 1211 genes in *L. cuprina* and 2502 genes in *C. hominivorax*, which may play an important role in parasitism. Further analysis of 1707 genes in *W. magnifica* revealed that 1548 (90.69%) of the genes could be annotated in the NR database. Of 1548 genes, 973 genes were annotated as experimentally uncharacterized genes. Analysis of the remaining 575 genes with specific functional annotations found some genes of interest in parasitism, such as olfactory‐related genes for site search for laying the larvae, insecticide resistance‐related genes and protease for digestion of host tissues (Data S1).

### 3.5 Gene family expansion and contraction of *W. magnifica*

The expansion and contraction of a species' gene family is often associated with its adaptive evolution. In the genome of *W. magnifica*, we identified 173 expanded gene families, comprising 2008 genes, and eight contracted families. Subsequently, a GO enrichment study of expanded gene families revealed 24 GO terms for cellular component categories, 10 GO terms for molecular function categories and 117 GO terms for the biological process categories (Figure 3b). We deemed the following GO terms of special interest as these terms likely help to improve the understanding of adaptation of *W. magnifica* to parasitic life‐style: structural constituent of chitin‐based cuticle (GO:0005214, *p*.adjust = 1.17e^−52^); structural constituent of cuticle (GO:0042302, *p*.adjust = 6.96e^−51^); cuticle development (GO:0042335, *p*.adjust = 1.10e^−35^); response to insecticide (GO:0017085, *p*.adjust = 4.23e^−21^); response to toxic substance (GO:0009636, *p*.adjust = 8.57e^−15^); response to bacterium (GO:0009617, *p*.adjust = 6.69e^−12^); defence response to bacterium (GO:0042742, *p*.adjust = 1.91e^−09^) and instar larval development (GO:0002168, *p*.adjust = 1.27e^−07^) (Figure 3b, Table S5). In addition, we also found that many GO terms are involved in transcription and chromatin modification, such as transcription elongation factor complex (GO:0008023, *p*.adjust = 6.44e^−34^); regulation of DNA‐templated transcription, elongation (GO:0032784, *p*.adjust = 9.12e^−31^); positive regulation of chromatin modification (GO:1903310, *p*.adjust = 4.21e^−20^) and positive regulation of histone modification (GO:0031058, *p*.adjust = 2.43e^−20^).

### 3.6 Genes under positive selection in *W. magnifica*

To determine genes potentially under positive selection in *W. magnifica*, 2045 single‐copy orthologues between *W. magnifica* and the other nine dipterous flies were multiply protein‐aligned and back‐translated. After this, we discarded 61 orthologues due to poorly aligned positions and retained 1984 orthologues, which were subjected to the branch‐site analysis. In the *W. magnifica* branch, 45 genes were identified as likely under positive selection. However, the annotated function of *D. melanogaster* orthologues of 13 of these genes was unknown. We investigated the functions of the remaining 32 genes, showing that the function of these genes spans a wide range of areas. Of these genes, the following genes are of particular interest: development‐related genes, including neuronal development (*Plex A and D*), muscle development (*Wnt2* and *Kon‐tiki*) and eye development (*Myt1*), Ca(2+) regulation (*RyR*), regulation of metabolism (*Wdr24*) and melanic pigmentation (*yellow‐k*) (Table S6).

## 4 DISCUSSION

### 4.1 De novo genome assembly from a single *W. magnifica* female using a low‐input DNA workflow

Genomic resources are essential for a thorough understanding of the molecular biology of an organism and the evolution of a species. In addition, they can provide insights into specific mechanisms of interest, for example in relation to environmental adaption, production traits in livestock, or for designing new control strategies of invasive species, for example, the spotted flesh fly (*W. magnifica*). Considering the small size of a single individual and the difficulty of obtaining inbreed of *W. magnifica*, we exploit the advantages of low DNA input and highly accurate HiFi sequence when using the low‐input DNA workflow for HiFi library preparation and sequencing on Pacbio sequel II with CCS mode. As a result, the de novo assembled genome consists of only 543 scaffolds with a scaffold N50 of 5.00 Mb. We used BUSCO and other metrics to assess the quality of the assembly and the results suggested that the de novo genome is of high quality, only with a few genes missing. In addition, our assembled genome is comparable to other genomes sequenced using various strategies such as *L. cuprina* (Anstead et al., 2015), *C. hominivorax* (Scott et al., 2020) or *S. peregrina* (Ren et al., 2021), both in terms of N50 and BUSCO results. Therefore, the strategy used in this study can serve as a reference sequencing approach for some dipterous flies that are small and not easy to rear in the lab. This also can save time for flies which can be reared in the lab but are challenging to inbreed. Importantly, this strategy will facilitate further insect sequencing projects like the 5000 Insect Genome Project (i5k) (i5K Consortium, 2013). Although our currently assembled genome has a very high quality, which can meet the requirements for genomic applications in *W. magnifica's* pest control, such as the development of Cas9‐based homing gene drives strains (Hammond et al., 2016) and transgenic sexing strains (Concha et al., 2020; Li et al., 2014), we suggest using Hi‐C technology to capture the organizational structure of chromatin in three dimensions. This will enhance further analysis, such as identification of promoter‐enhancer interactions for gene regulation studies and detection of structural rearrangements.

### 4.2 Parasitism‐related genes, gene families of expansion and positively selected genes related to adaptation and evolution in *W. magnifica*

Myiasis is known as a disease of living vertebrates invaded by dipteran larvae, which is of great medical and veterinary importance, as it affects not only wild and domestic animals but also humans in developed and developing countries worldwide (Zumpt, 1965). Based on the dependence degree to host, myiasis can be classified into three types: accidental, facultative or obligatory myiasis (Scholl et al., 2019). In this study, to identify genes associated with parasitism we selected three obligatory myiasis‐causing flies, *C. hominivorax*, *L. cuprina* and *W. magnifica*, and one nonmyiasis‐causing fly, *D. melanogaster*. A number of parasitism‐related genes were identified, such as olfactory‐related genes, proteases and some insecticide‐resistant genes. Usually, myiasis‐causing flies use their olfactory system to detect the odour from the host's open wound or genitalia while looking for a site to lay their eggs or larvae. Thus, these olfactory genes, such as odorant receptor and odorant‐binding protein, may be involved in the behaviour of the search for egg‐laying sites. Once these larvae have been oviposited into the host, these proteases help the larvae digest the host's tissues into small molecule peptides and amino acids for development. In addition, we also found several insecticide‐resistant genes in the gene sets, especially cytochrome P450 (CYP450), which may confer the resistance of myiasis‐causing flies to insecticide. For example, *L. cuprina* has developed resistance to organochlorines (e.g., dieldrin/aldrin), organophosphates (OP) (e.g., diazinon), carbamate (e.g., butacarb) and others (Sandeman et al., 2014). Although we found many genes of interest in this gene set, there are still many genes that are defined as hypothetical proteins with little to no experimental evidence for their function or being characterized by a low identity to proteins with known function. However, these genes may play an essential role in parasitism of myiasis‐causing flies.

We also investigated which gene families expanded and which GO categories were enriched by these expanding families. This may enhance our understanding of the adaption of *W. magnifica* to its parasitic lifestyle and may help identify potential strategies for pest control. For example, from late April or early May to mid‐October in China, as *W. magnifica* infects its hosts, large quantities of insecticides are used to kill the larvae of *W. magnifica*. This likely leads to adaptation in *W. magnifica's* response to insecticide, as the GO terms “response to insecticide” and “response to toxic substance” are enriched. The body temperature of camels varies considerably, from 40°C during the day to 34°C at night (Schmidt‐Nielsen et al., 1956). Consequently, this may induce thermal stress in *W. magnifica* larvae. This might explain the expansion of gene families with GO terms such as “response to temperature stimulus”, “cellular response to heat” and “response to heat”. Additionally, bacteria grow on the wound of the infected host and the host's immune response may affect the larval environment. This may in part explain the GO enrichment results also identified a number of expanded gene families with genes enriched for infection response‐related terms, for example, “response to bacterium”, “defence response to bacterium”, “defence response to other organism”, “mucosal immune response” and “organ or tissue specific immune response”. The cuticle, acting as a barrier between living tissues and the surrounding atmosphere, is a multilayered structure, which has various functions, for example, the determination of the shape and appearance, insecticide resistance and constituting a physical barrier to prevent pathogen entry (Andersen, 1979; Balabanidou et al., 2018; Moussian, 2010). The current study found that gene families that expanded most were associated with several GO categories (according to *p*‐value) linked to cuticle development. These results indicate that the cuticle may play a very important role in the adaptation of *W. magnifica* to its parasitic lifestyle. Interestingly, we also found that many of the GO categories are involved in transcription and chromatin modification, which is responsible for the regulation of gene expression, suggesting there might have been some major changes in gene expression during the evolution of *W. magnifica's* parasitic lifestyle.

In this study, we obtained up to 45 positively selected genes with diverse functions and molecular processes. These genes subjected to positive selection are likely to contribute to *W. magnifica's* evolution and adaptation. For *W. magnifica*, the developmental stage of the embryo and larvae is inside the female fly and inside the open wound or genitalia of the host, respectively. Of positively selected genes, we found several genes associated with development, including neuronal development (*Plex A and D*) (Junqueira Alves et al., 2019; Overton et al., 2002; Soriano & Russell, 1998), muscle development (*Wnt2* and *Kon‐tiki*) (Estrada et al., 2007; Schnorrer et al., 2007) and eye development (*Myt1*) (Price et al., 2002). These genes may contribute to the adaptation of *W. magnifica's* embryo and larvae to in vivo development. Insects are able to find the location of food, mates, and egg‐laying sites with the help of their olfactory systems (De Bruyne & Baker, 2008; He et al., 2019). In *Drosophila*, interfering with *RyR* expression resulted in a defective olfactory behaviour in flies (Murmu et al., 2010). Therefore, this gene might have an essential role in *W. magnifica's* response to several odours from the host's wounds or genitalia during the search for egg‐laying sites. *Wdr24*, a component of a multiprotein GATOR2 complex, is a critical part of the cellular metabolism, such as nutrients in different species, including *Drosophila*. During the development of *W. magnifica*'s larvae, the nutrients are mainly derived from the host tissues. Perhaps *Wdr24* plays an important role in tissue metabolism (Cai et al., 2016; Kim, Nam, et al., 2019; Kim, Paggi, et al., 2019). *Drosophila's* melanic pigmentation in the wings, abdomen and thorax is now recognized to be related to the *yellow* locus (Ferguson et al., 2011). Compared to other myiasis‐causing flies, the abdomen of *W. magnifica* has distinguishing dark‐coloured spots, which might be associated with *yellow‐k*.

### 4.3 Potential applications for the control of *W. magnifica*

Although the larvae of *W. magnifica* parasitize several warm‐blooded vertebrates, in China, its primary host is camels. During the summer months when *W. magnifica's* populations are high, grazing Bactrian camels are present across the Gobi Desert or grassland, and therefore are not frequently inspected, resulting in infected camels not being treated in a timely manner and aggravating the infection. The severe infection in this condition poses a threat to important animal welfare and health of Bactrian camels, and induces especially reproduction problems, such as abortion. Unfortunately, the infection also affects the wild camel (*Camelus ferus*), which is listed as Critically Endangered by the International Union for the Conservation of Nature (IUCN). It is estimated that there are approximately 1000 individuals left, around 600 in the Gobi Desert in northwest China and probably only 450 at the Mongolian side. (https://www.wildcamels.com/). The threat to the wild camel can be especially devastating because *W. magnifica* is unmanageable in wildlife populations. Therefore, similar to Bactrian camel, vital research on the control of *W. magnifica* is important for the conservation of this critically endangered wild camel.

In contrast to *W. magnifica*, *C. hominivorax* was successfully eradicated from the USA and Central America by the application of the sterile insect technique (SIT) (Wyss, 2006). With regards to *W. magnifica*, the assembled genome could pave the path for the identification of reproduction‐related genes, which might contribute towards the development of further SIT (Baumhover et al., 1955; Knipling, 1955). However, for SIT or other methods of genetic control of *W. magnifica*, due to the current high mortality rate of rearing in the laboratory, this would require significant progress in methods for rearing *W. magnifica* on artificial diet. For the prevention and control of *W. magnifica* in camels and other livestock, vaccines might be an effective strategy. On the basis of the de novo assembled genome of *W. magnifica*, a great range of candidate vaccine antigens might be identified, and effective antigens likely involved in inducing a protective immune response of the infected host against *W. magnifica* at larval stages could be selected for the development of subunit vaccines in the future. So far, chemical control methods against *W. magnifica* infections dominate in China. However, the excessive use of insecticides can lead to insecticide resistance, as supported by our results from the analysis of expanded gene families. Genome‐guided identification is a comprehensive and promising strategy to screen new drug targets and discover new drugs (Olsen & Faergeman, 2012). The approach aims to identify candidate genes or gene products that can be inactivated by insecticides, without harming to the host animal. For investigation of gene functions and insecticidal target discovery, the RNA interference (RNAi), combined with the resulting phenotype, is an effective approach, because it has been a great success with many insects (Hu et al., 2016; Riga et al., 2020). For the evaluation of gene functions on a genome‐wide scale in *W. magnifica*, RNAi is not for routine use. In this case, essential single‐copy genes of *W. magnifica* can be predicted using functional genomic data (e.g., lethality) available for orthologues in *D. melanogaster*, for which potential insecticidal targets have already been identified (Anstead et al., 2015; Olsen & Faergeman, 2012). In addition, genome‐wide identification of complete chemosensory genes could likely be beneficial for suppressing the *W. magnifica* population and monitoring its behaviour by trapping flies by odours. Clearly, the de novo assembled genome has enabled us to enter an exciting era in which the door to the development or improvement of novel genetic, immunological and chemical control strategies for *W. magnifica* is opened.

In conclusion, we successfully assembled de novo the genome of *W. magnifica* using only one female adult individual. This assembled genome is 753.99 Mb in size with a scaffold N50 length of 5.00 Mb and 59.71% repeat elements. The RNA‐seq mapping rate and BUSCO scores indicate that the assembled genome is complete (93.62% overall RNA‐seq alignment rate and 98.8% complete BUSCOs found). In addition, 16,718 genes and 20,017 mRNA were predicted in the assembled genome; of these, 64.98% of genes can be functionally annotated in the UniProt/Sprot database. Phylogenetic analysis revealed that *W. magnifica* is most closely related to *S. bullata*, followed by *L. cuprina*. GO enrichment analysis showed that many of the expanded gene families contained genes annotated for immunity, insecticide‐resistance mechanisms, heat stress response and cuticle development, while positively selected genes displayed diverse functions. Clearly, the availability of the current *W. magnifica* genome resource lays a solid foundation for being able to address key biological questions and to facilitate the development of new prevention and control methods of this mammal's pest in the future.

## AUTHOR CONTRIBUTIONS

Zhipeng Jia and Pamela A. Burger conceived the project and received funding. Zhipeng Jia performed the genome annotation and comparative genomic analysis and wrote the first draft of the manuscript. Surong Hasi and Claus Vogl contributed new reagents, samples or analytical support. Pamela A. Burger, Surong Hasi and Claus Vogl supervised the project. All authors provided valuable advice, reviewed and approved the final manuscript.

## CONFLICT OF INTEREST

The authors declare no conflict of interest.

### OPEN RESEARCH BADGES

This article has earned an Open Data badge for making publicly available the digitally‐shareable data necessary to reproduce the reported results. The data is available at https://doi.org/10.5061/dryad.qfttdz0j8.

## BENEFITS‐SHARING STATEMENT

A research collaboration was developed with scientists from the countries providing genetic samples. All collaborators are included as coauthors. Benefits from this research accrue from the sharing of our data and results on public databases as described above.

## Supporting information


Data S1

Table S1‐S6

## Data Availability

The genome assembly of *W. magnifica* was deposited as a BioProject under accession number PRJNA778059. The PacBio HiFi sequence reads are deposited at NCBI under accession number: SRR16848117. The transcriptome data have been deposited in SRA, including 3 first stage larvae (SRR18178228, SRR18178229, SRR18178230), 3 second stage larvae (SRR18178225, SRR18178226, SRR18178227) and 3 adult flies (SRR18178222, SRR18178223, SRR18178224). In addition, the assembly and annotation of the W. magnifica genome are also available on Dryad (https://doi.org/10.5061/dryad.qfttdz0j8).
