# Supplementary material for: Genomic insights into evolution and control of Wohlfahrtia magnifica, a widely distributed myiasis‐causing fly of warm‐blooded vertebrates
Source: Mol Ecol Resour. 2022 Jun 18;22(7):2744–57. doi: 10.1111/1755-0998.13654 (PMC9545800; doi:10.1111/1755-0998.13654)
Supplement: Supplementary file 2 — Table S1‐S6 [file MEN-22-2744-s001.docx]

**Supplemental Information for:**

**Genomic insights into evolution and control of *Wohlfahrtia magnifica*, a widely distributed myiasis-causing fly of warm-blooded vertebrates**

Zhipeng Jia^1^, Surong Hasi^2^, Claus Vogl^3^, Pamela A. Burger^1^

**Table of Contents:**

| **Supplementary Table 1** | Page 2 |
| --- | --- |
| **Supplementary Table 2** | Page 3 |
| **Supplementary Table 3** | Page 4 |
| **Supplementary Table 4** | Page 5 |
| **Supplementary Table 5** | Page 6-14 |
| **Supplementary Table 6** | Page 15-17 |

**Supplementary Table 1.** Detection results of DNA sample for low input library preparation

| Sample | Fluorescence concentration (ng/μl) | UV concentration (ng/μl) | Volume(μl) | OD260/280 | OD260/230 | Total amount (μg) |
| --- | --- | --- | --- | --- | --- | --- |
| A single adult *W. magnifica* | 27 | 30.10 | 45 | 1.77 | 2.11 | 1.215 |

**Supplementary Table 2.** BUSCO analyses of *W. magnifica* and other nine dipterous flies based on diptera_odb10 gene set (number of BUSCOs: 3285). Genome: assessment of genome assembly completeness; gene set: assessment of predicted gene set completeness.

| Species | Mode | Complete (Single Copy) | | Complete (Duplicated) | | Fragmented | | Missing | |
| --- | --- | --- | --- | --- | --- | --- | --- | --- | --- |
|  |  | Percentage | Numbers | Percentage | Numbers | Percentage | Numbers | Percentage | Numbers |
| *W. magnifica* | Genome | 98.2 | 3,226 | 0.6 | 19 | 0.6 | 19 | 0.6 | 21 |
|  | Gene set | 81.9 | 2,691 | 15.3 | 501 | 1.3 | 42 | 1.5 | 51 |
| *A.aegypti* | Genome | 94.4 | 3,101 | 3.1 | 101 | 1.1 | 36 | 1.4 | 47 |
|  | Gene set | 60.5 | 1,989 | 38.9 | 1,278 | 0.2 | 7 | 0.5 | 11 |
| *A. gambiae* | Genome | 97.5 | 3,204 | 1.7 | 56 | 0.1 | 4 | 0.7 | 21 |
|  | Gene set | 87.5 | 2,874 | 11.6 | 381 | 0.4 | 13 | 0.5 | 17 |
| *D. melanogaster* | Genome | 99.0 | 3,252 | 0.4 | 14 | 0.2 | 5 | 0.4 | 14 |
|  | Gene set | 41.8 | 1,372 | 58.2 | 1,913 | 0.0 | 0 | 0.0 | 0 |
| *G. morsitans* | Genome | 96.7 | 3,176 | 1.3 | 43 | 1.0 | 32 | 1.0 | 34 |
|  | Gene set | 89.8 | 2,949 | 2.3 | 75 | 1.9 | 61 | 6.0 | 200 |
| *L. cuprina* | Genome | 96.2 | 3,159 | 1.6 | 53 | 0.9 | 31 | 1.3 | 42 |
|  | Gene set | 87.5 | 2,876 | 1.7 | 56 | 1.2 | 41 | 9.6 | 312 |
| *M. destructor* | Genome | 85.3 | 2,803 | 1.4 | 45 | 4.3 | 142 | 9.0 | 295 |
|  | Gene set | 71.1 | 2,334 | 13.6 | 448 | 4.4 | 146 | 10.9 | 357 |
| *M. domestica* | Genome | 94.9 | 3,116 | 2.2 | 71 | 1.2 | 39 | 1.7 | 59 |
|  | Gene set | 73.2 | 2,406 | 25.7 | 845 | 0.8 | 25 | 0.3 | 9 |
| *S. bullata* | Genome | 86.7 | 2,847 | 0.8 | 27 | 6.8 | 225 | 5.7 | 186 |
|  | Gene set | 77.9 | 2560 | 1.1 | 35 | 8.7 | 286 | 12.3 | 404 |
| *S. calcitrans* | Genome | 96.2 | 3,159 | 0.9 | 29 | 1.1 | 37 | 1.8 | 60 |
|  | Gene set | 67.7 | 2,223 | 31.6 | 1,039 | 0.5 | 15 | 0.2 | 8 |

**Supplementary Table 3.** Comparison of summary statistics of repeat element of *W. magnifica* and other dipterous flies

| Repeat element | *W. magnifica* | | | *C. hominivorax* | | | *L. cuprina* | | | *S. bullata* | | |
| --- | --- | --- | --- | --- | --- | --- | --- | --- | --- | --- | --- | --- |
|  | Numbers | Bases | % of Genome | Numbers | Bases | % of Genome | Numbers | Bases | % of Genome | Numbers | Bases | % of Genome |
| SINEs | 0 | 0 | 0.00 | 63 | 5,637 | 0.00 | 434 | 64,889 | 0.01 | 33,478 | 7,221,604 | 1.52 |
| LINEs | 313,495 | 97,444,031 | 12.92 | / | 12,057,930 | 2.26 | 61,619 | 15,766,996 | 3.35 | 179,696 | 32,921,812 | 6.91 |
| LTR | 98,752 | 29,004,031 | 3.85 | / | 2,848,352 | 0.53 | 16,688 | 5,901,984 | 1.25 | 17,235 | 6,433,260 | 1.35 |
| DNA | 473,914 | 125,334,368 | 16.62 | / | 36,993,232 | 6.93 | 60,359 | 12,837,946 | 2.73 | 127,299 | 19,969,616 | 4.19 |
| Unclassified | 976,699 | 176,211,233 | 23.37 | 669,162 | 135,021,258 | 25.28 | 383,500 | 78,482,695 | 16.68 | 397,153 | 59,304,762 | 12.45 |
| Simple repeats | 279,951 | 18,690,856 | 2.48 | 320,870 | 17,432,637 | 3.26 | 150,125 | 24,344,465 | 5.17 | 416,660 | 18,229,578 | 3.83 |
| Low complexity | 61,088 | 2,981,425 | 0.40 | 73,844 | 3,798,472 | 0.71 | 370,031 | 18,958,378 | 4.03 | 89,350 | 4,553,468 | 0.96 |
| Total | / | 450,243,625 | 59.71 | 1,467,371 | 241,520,001 | 45.22 | 1,058,216 | 272,180,260 | 57.82 | / | 148,389,050 | 31.15 |

**Supplementary Table 4.** Distribution of genes in *W. magnifica* and other dipterous flies. 1:1 orthologs: single-copy orthologs; N:N orthologs: multiple-copy orthologs; species-specific orthologs: present in specific species including multi copy and single copy; other orthologs: the remaining orthologs

| Type | | *A. aegypti* | *A. gambiae* | *D. melanogaster* | *G. morsitans* | *L. cuprina* | *M. destructor* | *M. domestica* | *S. bullata* | *S. calcitrans* | *W. magnifica* |
| --- | --- | --- | --- | --- | --- | --- | --- | --- | --- | --- | --- |
| Number of genes | | 14,718 | 13,094 | 13,968 | 12,494 | 14,452 | 19,927 | 14,402 | 15,763 | 14,078 | 16,718 |
| 1:1 orthologs | | 2,045 | 2,045 | 2,045 | 2,045 | 2,045 | 2,045 | 2,045 | 2,045 | 2,045 | 2,045 |
| N:N orthologs | | 5,240 | 4,442 | 4,604 | 4,725 | 4,352 | 4,544 | 5,137 | 5,199 | 4,895 | 4,972 |
| Species-specific orthologs | Multi copy | 1,029 | 834 | 707 | 402 | 723 | 4,259 | 317 | 501 | 289 | 943 |
|  | Single copy | 456 | 563 | 1261 | 1,099 | 2,048 | 5,066 | 319 | 2,459 | 320 | 793 |
| Other orthologs | | 5,948 | 5,210 | 5,351 | 4,223 | 5,284 | 4,013 | 6,584 | 5,559 | 6,529 | 7,965 |

**Supplementary Table 5.** GO classification of expanded gene families of *W. magnifica*

| GO_ID | GO_class | GO_description | Pvalue | Qvalue | Gene Numbers | Reference  Numbers | RichFactor |
| --- | --- | --- | --- | --- | --- | --- | --- |
| GO:0005214 | Molecular Function | structural constituent of chitin-based cuticle | 1.14E-54 | 1.17E-52 | 73 | 140 | 0.521 |
| GO:0042302 | Molecular Function | structural constituent of cuticle | 1.37E-52 | 6.96E-51 | 74 | 152 | 0.487 |
| GO:0005576 | Cellular Component | extracellular region | 9.71E-42 | 1.10E-39 | 95 | 453 | 0.21 |
| GO:0044421 | Cellular Component | extracellular region part | 3.79E-40 | 2.14E-38 | 88 | 400 | 0.22 |
| GO:0042335 | Biological Process | cuticle development | 1.61E-38 | 1.10E-35 | 73 | 202 | 0.361 |
| GO:0008023 | Cellular Component | transcription elongation factor complex | 1.71E-35 | 6.44E-34 | 38 | 65 | 0.585 |
| GO:0005198 | Molecular Function | structural molecule activity | 3.97E-33 | 1.35E-31 | 84 | 337 | 0.249 |
| GO:0001824 | Biological Process | blastocyst development | 8.40E-34 | 2.89E-31 | 27 | 27 | 1 |
| GO:0000785 | Cellular Component | chromatin | 1.03E-32 | 2.91E-31 | 56 | 186 | 0.301 |
| GO:0032784 | Biological Process | regulation of DNA-templated transcription, elongation | 3.98E-33 | 9.12E-31 | 38 | 57 | 0.667 |
| GO:0031062 | Biological Process | positive regulation of histone methylation | 2.13E-29 | 3.66E-27 | 31 | 42 | 0.738 |
| GO:0018023 | Biological Process | peptidyl-lysine trimethylation | 1.25E-26 | 1.72E-24 | 27 | 35 | 0.771 |
| GO:0044427 | Cellular Component | chromosomal part | 1.25E-24 | 2.81E-23 | 59 | 288 | 0.205 |
| GO:0032786 | Biological Process | positive regulation of DNA-templated transcription, elongation | 1.15E-24 | 1.32E-22 | 28 | 42 | 0.667 |
| GO:0001701 | Biological Process | in utero embryonic development | 1.65E-24 | 1.62E-22 | 27 | 39 | 0.692 |
| GO:0031060 | Biological Process | regulation of histone methylation | 2.57E-23 | 2.21E-21 | 31 | 57 | 0.544 |
| GO:0060795 | Biological Process | cell fate commitment involved in formation of primary germ layer | 2.17E-22 | 1.66E-20 | 27 | 44 | 0.614 |
| GO:0006354 | Biological Process | DNA-templated transcription, elongation | 3.50E-22 | 2.41E-20 | 38 | 95 | 0.4 |
| GO:0031058 | Biological Process | positive regulation of histone modification | 3.89E-22 | 2.43E-20 | 31 | 61 | 0.508 |
| GO:1903310 | Biological Process | positive regulation of chromatin modification | 7.35E-22 | 4.21E-20 | 31 | 62 | 0.5 |
| GO:2001252 | Biological Process | positive regulation of chromosome organization | 2.50E-21 | 1.32E-19 | 31 | 64 | 0.484 |
| GO:0017085 | Biological Process | response to insecticide | 4.23E-21 | 2.08E-19 | 25 | 40 | 0.625 |
| GO:0001704 | Biological Process | formation of primary germ layer | 3.71E-18 | 1.70E-16 | 27 | 58 | 0.466 |
| GO:0018022 | Biological Process | peptidyl-lysine methylation | 4.10E-18 | 1.76E-16 | 28 | 63 | 0.444 |
| GO:0043414 | Biological Process | macromolecule methylation | 5.43E-17 | 2.20E-15 | 38 | 128 | 0.297 |
| GO:0016571 | Biological Process | histone methylation | 1.98E-16 | 7.16E-15 | 31 | 88 | 0.352 |
| GO:0031056 | Biological Process | regulation of histone modification | 1.98E-16 | 7.16E-15 | 31 | 88 | 0.352 |
| GO:0005694 | Cellular Component | chromosome | 3.97E-16 | 7.47E-15 | 59 | 421 | 0.14 |
| GO:0032259 | Biological Process | methylation | 3.92E-16 | 1.35E-14 | 38 | 135 | 0.281 |
| GO:0006325 | Biological Process | chromatin organization | 6.40E-16 | 2.10E-14 | 67 | 379 | 0.177 |
| GO:0043566 | Molecular Function | structure-specific DNA binding | 9.93E-16 | 2.53E-14 | 31 | 98 | 0.316 |
| GO:1902275 | Biological Process | regulation of chromatin organization | 1.62E-15 | 5.06E-14 | 34 | 113 | 0.301 |
| GO:0044451 | Cellular Component | nucleoplasm part | 3.32E-15 | 5.36E-14 | 49 | 316 | 0.155 |
| GO:0005654 | Cellular Component | nucleoplasm | 3.83E-15 | 5.41E-14 | 50 | 329 | 0.152 |
| GO:0010628 | Biological Process | positive regulation of gene expression | 1.97E-15 | 5.90E-14 | 41 | 163 | 0.252 |
| GO:0006479 | Biological Process | protein methylation | 2.35E-15 | 6.74E-14 | 31 | 95 | 0.326 |
| GO:0014031 | Biological Process | mesenchymal cell development | 4.87E-15 | 1.34E-13 | 23 | 52 | 0.442 |
| GO:0048762 | Biological Process | mesenchymal cell differentiation | 8.12E-15 | 2.15E-13 | 23 | 53 | 0.434 |
| GO:0009636 | Biological Process | response to toxic substance | 8.57E-15 | 2.18E-13 | 26 | 69 | 0.377 |
| GO:0008213 | Biological Process | protein alkylation | 2.18E-14 | 5.37E-13 | 31 | 102 | 0.304 |
| GO:0033044 | Biological Process | regulation of chromosome organization | 2.38E-14 | 5.65E-13 | 37 | 144 | 0.257 |
| GO:1903308 | Biological Process | regulation of chromatin modification | 2.95E-14 | 6.76E-13 | 31 | 103 | 0.301 |
| GO:0008593 | Biological Process | regulation of Notch signaling pathway | 3.97E-14 | 8.81E-13 | 31 | 104 | 0.298 |
| GO:0007369 | Biological Process | gastrulation | 7.17E-13 | 1.54E-11 | 28 | 94 | 0.298 |
| GO:0048864 | Biological Process | stem cell development | 1.06E-12 | 2.21E-11 | 24 | 70 | 0.343 |
| GO:0045893 | Biological Process | positive regulation of transcription, DNA-templated | 1.35E-12 | 2.59E-11 | 29 | 103 | 0.282 |
| GO:1902680 | Biological Process | positive regulation of RNA biosynthetic process | 1.35E-12 | 2.59E-11 | 29 | 103 | 0.282 |
| GO:1903508 | Biological Process | positive regulation of nucleic acid-templated transcription | 1.35E-12 | 2.59E-11 | 29 | 103 | 0.282 |
| GO:0060485 | Biological Process | mesenchyme development | 1.48E-12 | 2.75E-11 | 23 | 65 | 0.354 |
| GO:0051254 | Biological Process | positive regulation of RNA metabolic process | 2.18E-12 | 3.96E-11 | 31 | 119 | 0.261 |
| GO:0007219 | Biological Process | Notch signaling pathway | 5.64E-12 | 9.95E-11 | 31 | 123 | 0.252 |
| GO:0009617 | Biological Process | response to bacterium | 6.69E-12 | 1.15E-10 | 52 | 305 | 0.17 |
| GO:0043009 | Biological Process | chordate embryonic development | 4.77E-11 | 8.01E-10 | 28 | 110 | 0.255 |
| GO:0006259 | Biological Process | DNA metabolic process | 7.78E-11 | 1.27E-09 | 65 | 460 | 0.141 |
| GO:0042742 | Biological Process | defense response to bacterium | 1.19E-10 | 1.91E-09 | 39 | 205 | 0.19 |
| GO:0010557 | Biological Process | positive regulation of macromolecule biosynthetic process | 1.60E-10 | 2.50E-09 | 29 | 123 | 0.236 |
| GO:0010638 | Biological Process | positive regulation of organelle organization | 1.67E-10 | 2.55E-09 | 31 | 139 | 0.223 |
| GO:0045935 | Biological Process | positive regulation of nucleobase-containing compound metabolic process | 1.97E-10 | 2.95E-09 | 32 | 148 | 0.216 |
| GO:0051173 | Biological Process | positive regulation of nitrogen compound metabolic process | 4.87E-10 | 7.13E-09 | 32 | 153 | 0.209 |
| GO:0031328 | Biological Process | positive regulation of cellular biosynthetic process | 6.56E-10 | 9.40E-09 | 29 | 130 | 0.223 |
| GO:0014074 | Biological Process | response to purine-containing compound | 1.43E-09 | 2.01E-08 | 14 | 32 | 0.438 |
| GO:0098542 | Biological Process | defense response to other organism | 1.58E-09 | 2.17E-08 | 39 | 223 | 0.175 |
| GO:0051276 | Biological Process | chromosome organization | 4.88E-09 | 6.58E-08 | 67 | 530 | 0.126 |
| GO:0002385 | Biological Process | mucosal immune response | 6.35E-09 | 8.40E-08 | 9 | 13 | 0.692 |
| GO:0009891 | Biological Process | positive regulation of biosynthetic process | 1.55E-08 | 2.02E-07 | 29 | 148 | 0.196 |
| GO:0018205 | Biological Process | peptidyl-lysine modification | 1.73E-08 | 2.20E-07 | 28 | 140 | 0.2 |
| GO:0000786 | Cellular Component | nucleosome | 3.37E-08 | 3.81E-07 | 7 | 10 | 0.7 |
| GO:0044815 | Cellular Component | DNA packaging complex | 3.37E-08 | 3.81E-07 | 7 | 10 | 0.7 |
| GO:0048863 | Biological Process | stem cell differentiation | 3.19E-08 | 3.99E-07 | 24 | 110 | 0.218 |
| GO:0031012 | Cellular Component | extracellular matrix | 5.05E-08 | 5.18E-07 | 17 | 80 | 0.212 |
| GO:0090304 | Biological Process | nucleic acid metabolic process | 4.48E-08 | 5.50E-07 | 166 | 1884 | 0.088 |
| GO:0051707 | Biological Process | response to other organism | 7.46E-08 | 9.01E-07 | 53 | 405 | 0.131 |
| GO:0043207 | Biological Process | response to external biotic stimulus | 8.80E-08 | 1.04E-06 | 53 | 407 | 0.13 |
| GO:0009607 | Biological Process | response to biotic stimulus | 1.22E-07 | 1.42E-06 | 53 | 411 | 0.129 |
| GO:0002168 | Biological Process | instar larval development | 1.27E-07 | 1.46E-06 | 10 | 21 | 0.476 |
| GO:0009952 | Biological Process | anterior/posterior pattern specification | 2.02E-07 | 2.27E-06 | 23 | 112 | 0.205 |
| GO:0000122 | Biological Process | negative regulation of transcription from RNA polymerase II promoter | 2.20E-07 | 2.44E-06 | 10 | 22 | 0.455 |
| GO:0002251 | Biological Process | organ or tissue specific immune response | 3.27E-07 | 3.58E-06 | 9 | 18 | 0.5 |
| GO:0031401 | Biological Process | positive regulation of protein modification process | 4.87E-07 | 5.23E-06 | 36 | 242 | 0.149 |
| GO:0016568 | Biological Process | chromatin modification | 1.12E-06 | 1.18E-05 | 41 | 304 | 0.135 |
| GO:0034605 | Biological Process | cellular response to heat | 1.27E-06 | 1.33E-05 | 6 | 8 | 0.75 |
| GO:0043228 | Cellular Component | non-membrane-bounded organelle | 3.92E-06 | 3.37E-05 | 100 | 1507 | 0.066 |
| GO:0043232 | Cellular Component | intracellular non-membrane-bounded organelle | 3.92E-06 | 3.37E-05 | 100 | 1507 | 0.066 |
| GO:0032993 | Cellular Component | protein-DNA complex | 4.17E-06 | 3.37E-05 | 7 | 17 | 0.412 |
| GO:0045892 | Biological Process | negative regulation of transcription, DNA-templated | 3.38E-06 | 3.42E-05 | 17 | 78 | 0.218 |
| GO:1903507 | Biological Process | negative regulation of nucleic acid-templated transcription | 3.38E-06 | 3.42E-05 | 17 | 78 | 0.218 |
| GO:0051130 | Biological Process | positive regulation of cellular component organization | 3.65E-06 | 3.64E-05 | 31 | 210 | 0.148 |
| GO:0015935 | Cellular Component | small ribosomal subunit | 6.56E-06 | 4.94E-05 | 10 | 40 | 0.25 |
| GO:0018193 | Biological Process | peptidyl-amino acid modification | 1.01E-05 | 9.95E-05 | 28 | 189 | 0.148 |
| GO:0003676 | Molecular Function | nucleic acid binding | 4.90E-06 | 0.000100047 | 120 | 1449 | 0.083 |
| GO:0010604 | Biological Process | positive regulation of macromolecule metabolic process | 1.36E-05 | 0.000131563 | 49 | 430 | 0.114 |
| GO:0032270 | Biological Process | positive regulation of cellular protein metabolic process | 1.41E-05 | 0.000135208 | 36 | 279 | 0.129 |
| GO:0051247 | Biological Process | positive regulation of protein metabolic process | 1.66E-05 | 0.000156402 | 36 | 281 | 0.128 |
| GO:0009792 | Biological Process | embryo development ending in birth or egg hatching | 1.80E-05 | 0.0001674 | 30 | 216 | 0.139 |
| GO:0006260 | Biological Process | DNA replication | 2.82E-05 | 0.000258821 | 19 | 109 | 0.174 |
| GO:0016569 | Biological Process | covalent chromatin modification | 3.09E-05 | 0.000276442 | 31 | 233 | 0.133 |
| GO:0016570 | Biological Process | histone modification | 3.09E-05 | 0.000276442 | 31 | 233 | 0.133 |
| GO:0006139 | Biological Process | nucleobase-containing compound metabolic process | 3.15E-05 | 0.000278039 | 167 | 2094 | 0.08 |
| GO:1902679 | Biological Process | negative regulation of RNA biosynthetic process | 4.56E-05 | 0.000397085 | 17 | 94 | 0.181 |
| GO:0035821 | Biological Process | modification of morphology or physiology of other organism | 5.65E-05 | 0.000486037 | 8 | 24 | 0.333 |
| GO:0034728 | Biological Process | nucleosome organization | 6.18E-05 | 0.000525241 | 13 | 61 | 0.213 |
| GO:0042594 | Biological Process | response to starvation | 8.42E-05 | 0.000706588 | 10 | 39 | 0.256 |
| GO:0046483 | Biological Process | heterocycle metabolic process | 0.000102238 | 0.000847468 | 167 | 2137 | 0.078 |
| GO:0006952 | Biological Process | defense response | 0.000110019 | 0.000901105 | 45 | 416 | 0.108 |
| GO:2000113 | Biological Process | negative regulation of cellular macromolecule biosynthetic process | 0.000149171 | 0.001207409 | 17 | 103 | 0.165 |
| GO:0006306 | Biological Process | DNA methylation | 0.000167211 | 0.00132231 | 7 | 21 | 0.333 |
| GO:0044728 | Biological Process | DNA methylation or demethylation | 0.000167211 | 0.00132231 | 7 | 21 | 0.333 |
| GO:0006323 | Biological Process | DNA packaging | 0.000203612 | 0.001591876 | 10 | 43 | 0.233 |
| GO:0006305 | Biological Process | DNA alkylation | 0.00031675 | 0.002421379 | 7 | 23 | 0.304 |
| GO:0006334 | Biological Process | nucleosome assembly | 0.00031675 | 0.002421379 | 7 | 23 | 0.304 |
| GO:0031981 | Cellular Component | nuclear lumen | 0.000344006 | 0.002429541 | 64 | 958 | 0.067 |
| GO:0030246 | Molecular Function | carbohydrate binding | 0.000156879 | 0.002666943 | 7 | 22 | 0.318 |
| GO:0006725 | Biological Process | cellular aromatic compound metabolic process | 0.00038624 | 0.002920147 | 167 | 2190 | 0.076 |
| GO:0048029 | Molecular Function | monosaccharide binding | 0.000210907 | 0.003073213 | 5 | 11 | 0.455 |
| GO:0043189 | Cellular Component | H4/H2A histone acetyltransferase complex | 0.000495155 | 0.003108475 | 6 | 24 | 0.25 |
| GO:1902562 | Cellular Component | H4 histone acetyltransferase complex | 0.000495155 | 0.003108475 | 6 | 24 | 0.25 |
| GO:0031514 | Cellular Component | motile cilium | 0.000644736 | 0.003834481 | 4 | 10 | 0.4 |
| GO:0070013 | Cellular Component | intracellular organelle lumen | 0.000700343 | 0.003904975 | 64 | 983 | 0.065 |
| GO:0044449 | Cellular Component | contractile fiber part | 0.000737676 | 0.003904975 | 11 | 80 | 0.138 |
| GO:0043233 | Cellular Component | organelle lumen | 0.000760261 | 0.003904975 | 64 | 986 | 0.065 |
| GO:0006304 | Biological Process | DNA modification | 0.000558147 | 0.00417397 | 7 | 25 | 0.28 |
| GO:0051253 | Biological Process | negative regulation of RNA metabolic process | 0.000573229 | 0.004240661 | 17 | 115 | 0.148 |
| GO:0033043 | Biological Process | regulation of organelle organization | 0.000703057 | 0.005145779 | 37 | 350 | 0.106 |
| GO:0031974 | Cellular Component | membrane-enclosed lumen | 0.001291911 | 0.006347214 | 64 | 1006 | 0.064 |
| GO:0043292 | Cellular Component | contractile fiber | 0.001360206 | 0.006404302 | 11 | 86 | 0.128 |
| GO:1901360 | Biological Process | organic cyclic compound metabolic process | 0.000899214 | 0.006493923 | 170 | 2273 | 0.075 |
| GO:0071824 | Biological Process | protein-DNA complex subunit organization | 0.000906129 | 0.006493923 | 13 | 79 | 0.165 |
| GO:0016779 | Molecular Function | nucleotidyltransferase activity | 0.00055632 | 0.007093077 | 12 | 70 | 0.171 |
| GO:0034641 | Biological Process | cellular nitrogen compound metabolic process | 0.001156246 | 0.008201002 | 173 | 2331 | 0.074 |
| GO:0009408 | Biological Process | response to heat | 0.001214106 | 0.008523522 | 8 | 36 | 0.222 |
| GO:0003677 | Molecular Function | DNA binding | 0.000902933 | 0.00994628 | 49 | 544 | 0.09 |
| GO:0016628 | Molecular Function | oxidoreductase activity, acting on the CH-CH group of donors, NAD or NADP as acceptor | 0.000975125 | 0.00994628 | 8 | 37 | 0.216 |
| GO:0010558 | Biological Process | negative regulation of macromolecule biosynthetic process | 0.001491014 | 0.010361791 | 17 | 125 | 0.136 |
| GO:0006357 | Biological Process | regulation of transcription from RNA polymerase II promoter | 0.001621179 | 0.01098685 | 52 | 564 | 0.092 |
| GO:0051262 | Biological Process | protein tetramerization | 0.001625076 | 0.01098685 | 6 | 22 | 0.273 |
| GO:0045934 | Biological Process | negative regulation of nucleobase-containing compound metabolic process | 0.001628864 | 0.01098685 | 17 | 126 | 0.135 |
| GO:0031497 | Biological Process | chromatin assembly | 0.001811402 | 0.012099465 | 7 | 30 | 0.233 |
| GO:0031327 | Biological Process | negative regulation of cellular biosynthetic process | 0.002108493 | 0.01394849 | 17 | 129 | 0.132 |
| GO:0006333 | Biological Process | chromatin assembly or disassembly | 0.002693413 | 0.01764827 | 7 | 32 | 0.219 |
| GO:0006366 | Biological Process | transcription from RNA polymerase II promoter | 0.003061846 | 0.019873114 | 52 | 581 | 0.09 |
| GO:0009890 | Biological Process | negative regulation of biosynthetic process | 0.003701546 | 0.023751898 | 17 | 136 | 0.125 |
| GO:0072319 | Biological Process | vesicle uncoating | 0.003728496 | 0.023751898 | 2 | 2 | 1 |
| GO:0050919 | Biological Process | negative chemotaxis | 0.004071389 | 0.025698307 | 6 | 26 | 0.231 |
| GO:0009893 | Biological Process | positive regulation of metabolic process | 0.004396204 | 0.027496257 | 49 | 550 | 0.089 |
| GO:0009266 | Biological Process | response to temperature stimulus | 0.004902755 | 0.030388247 | 15 | 117 | 0.128 |
| GO:0006406 | Biological Process | mRNA export from nucleus | 0.006016631 | 0.03663223 | 5 | 20 | 0.25 |
| GO:0071427 | Biological Process | mRNA-containing ribonucleoprotein complex export from nucleus | 0.006016631 | 0.03663223 | 5 | 20 | 0.25 |
| GO:0006342 | Biological Process | chromatin silencing | 0.007356202 | 0.043629889 | 7 | 38 | 0.184 |
| GO:0045814 | Biological Process | negative regulation of gene expression, epigenetic | 0.007356202 | 0.043629889 | 7 | 38 | 0.184 |
| GO:0065004 | Biological Process | protein-DNA complex assembly | 0.007356202 | 0.043629889 | 7 | 38 | 0.184 |
| GO:0043933 | Biological Process | macromolecular complex subunit organization | 0.00757468 | 0.044541705 | 80 | 1010 | 0.079 |

**Supplementary Table 6.** Genes with evidence of positive selection in genome of *W. magnifica*

| Gene.ID | 1. *Melanogaster*   orthologs | Annotated function of *D. melanogaster* orthologs | l0 | l1 | 2(l1-l0) | pvalue | p.adj |
| --- | --- | --- | --- | --- | --- | --- | --- |
| Woma_00015236-RA | FBgn0025741 | Gene=Plex A, Plexin A | -36626.655076 | -36696.497668 | 139.685 | 0.000e+00 | 0.000000e+00 |
| Woma_00006011-RB | FBgn0051158 | Gene=Efa6, Exchange factor for Arf 6 | -18461.556751 | -18487.733199 | 52.3528 | 4.637e-13 | 1.174707e-10 |
| Woma_00009437-RB | FBgn0041789 | Gene=Pax, Paxillin | -7267.258270 | -7289.974646 | 45.4328 | 1.580e-11 | 3.002000e-09 |
| Woma_00012634-RA | FBgn0004360 | Gene=Wnt2, Wnt oncogene analog 2 | -6392.824908 | -6410.287286 | 34.9248 | 3.427e-09 | 5.209040e-07 |
| Woma_00005680-RA | FBgn0037648 | Gene=CG11975 | -6113.242801 | -6128.605055 | 30.7246 | 2.974e-08 | 3.767067e-06 |
| Woma_00006399-RA | FBgn0035532 | Gene=CG15014 | -6957.565389 | -6972.300373 | 29.47 | 5.679e-08 | 6.165771e-06 |
| Woma_00007179-RA | FBgn0011286 | Gene=RyR, Ryanodine receptor | -79959.098831 | -79972.587268 | 26.9768 | 2.059e-07 | 1.956050e-05 |
| Woma_00010254-RA | FBgn0020251 | Gene=sfl, sulfateless | -13872.742861 | -13884.719202 | 23.9526 | 9.874e-07 | 8.338044e-05 |
| Woma_00004948-RA | FBgn0032901 | Gene=sky, skywalker | -6132.164444 | -6142.896530 | 21.4642 | 3.605e-06 | 2.689709e-04 |
| Woma_00000964-RA | FBgn0033482 | Gene=CG1371 | -28464.907561 | -28475.566048 | 21.317 | 3.893e-06 | 2.689709e-04 |
| Woma_00012285-RA | FBgn0033166 | Gene=Eaf, ELL-associated factor | -1396.137382 | -1406.312657 | 20.3506 | 6.447e-06 | 4.083100e-04 |
| Woma_00006976-RA | FBgn0000411 | Gene=D, Dichaete | -4800.598853 | -4810.275906 | 19.3541 | 1.086e-05 | 6.348923e-04 |
| Woma_00012332-RA | FBgn0053111 | Gene=CG33111 | -8013.737171 | -8022.924721 | 18.3751 | 1.814e-05 | 9.847429e-04 |
| Woma_00010202-RA | FBgn0040298 | Gene=Myt1, Myt1 | -10372.732751 | -10381.357864 | 17.2502 | 3.277e-05 | 1.660347e-03 |
| Woma_00010517-RA | FBgn0036462 | Gene=mRpL39, mitochondrial ribosomal protein L39 | -7331.936570 | -7339.708681 | 15.5442 | 8.060e-05 | 3.828500e-03 |
| Woma_00009906-RA | FBgn0030246 | Gene=CG1582 | -24202.940356 | -24210.037570 | 14.1944 | 1.649e-04 | 6.652000e-03 |
| Woma_00009502-RA | FBgn0031878 | Gene=sip2, septin interacting protein 2 | -11211.529651 | -11218.618818 | 14.1783 | 1.663e-04 | 6.652000e-03 |
| Woma_00004111-RA | FBgn0031628 | Gene=CG3294 | -11451.435441 | -11458.361536 | 13.8522 | 1.978e-04 | 7.516400e-03 |
| Woma_00009853-RA | FBgn0038272 | Gene=Dph2, Diphthamide biosynthesis 2 | -11121.555612 | -11128.296688 | 13.4822 | 2.408e-04 | 8.714667e-03 |
| Woma_00001951-RA | FBgn0259223 | Gene=CG42323 | -2276.150447 | -2282.501176 | 12.7015 | 3.654e-04 | 1.214017e-02 |
| Woma_00010582-RA | FBgn0264494 | Gene=CG17646 | -2363.568276 | -2369.913759 | 12.691 | 3.674e-04 | 1.214017e-02 |
| Woma_00009603-RA | FBgn0028484 | Gene=Ack, Activated Cdc42 kinase | -13925.010681 | -13931.286369 | 12.5514 | 3.959e-04 | 1.253683e-02 |
| Woma_00003792-RA | FBgn0037632 | Gene=CCT7, Chaperonin containing TCP1 subunit 7 | -4774.823590 | -4781.005339 | 12.3635 | 4.378e-04 | 1.330912e-02 |
| Woma_00005239-RA | FBgn0260940 | Gene=lsn, larsen | -4603.138134 | -4609.122887 | 11.9695 | 5.408e-04 | 1.580800e-02 |
| Woma_00010013-RA | FBgn0034816 | Gene=CG3085 | -9131.729534 | -9137.643070 | 11.8271 | 5.837e-04 | 1.643007e-02 |
| Woma_00005272-RA | FBgn0043455 | Gene=CG5986 | -4759.062292 | -4764.825344 | 11.5261 | 6.863e-04 | 1.798579e-02 |
| Woma_00008799-RB | FBgn0284408 | Gene=trol, terribly reduced optic lobes | -64876.210451 | -64881.897841 | 11.3748 | 7.445e-04 | 1.886067e-02 |
| Woma_00005324-RB | FBgn0260468 | Gene=CG7950 | -2446.441525 | -2452.015719 | 11.1484 | 8.410e-04 | 2.061806e-02 |
| Woma_00004396-RA | FBgn0027518 | Gene=Wdr24, WD repeat domain 24 | -15946.376831 | -15951.694069 | 10.6345 | 1.110e-03 | 2.556364e-02 |
| Woma_00015317-RA | FBgn0259994 | Gene=OtopLa, Otopetrin-like a | -5843.961134 | -5849.198485 | 10.4747 | 1.210e-03 | 2.671122e-02 |
| Woma_00007237-RB | FBgn0038830 | Gene=CG17272 | -2484.239895 | -2489.435794 | 10.3918 | 1.266e-03 | 2.671122e-02 |
| Woma_00005723-RA | FBgn0032683 | Gene=kon, Kon-tiki | -49093.394907 | -49098.542078 | 10.2943 | 1.334e-03 | 2.671122e-02 |
| Woma_00011309-RA | FBgn0027889 | Gene=ball, ballchen | -10865.829363 | -10870.950115 | 10.2415 | 1.373e-03 | 2.671122e-02 |
| Woma_00006898-RA | FBgn0036504 | Gene=yellow-k, yellow-k | -7672.990773 | -7678.091869 | 10.2022 | 1.403e-03 | 2.671122e-02 |
| Woma_00005570-RA | FBgn0039688 | Gene=Kul, Kuzbanian-like | -14059.741661 | -14064.838534 | 10.1937 | 1.409e-03 | 2.671122e-02 |
| Woma_00000912-RA | FBgn0086359 | Gene=Invadolysin, Invadolysin | -10880.476650 | -10885.566905 | 10.1805 | 1.419e-03 | 2.671122e-02 |
| Woma_00007702-RA | FBgn0003416 | Gene=sl, small wing | -11335.580908 | -11340.657219 | 10.1526 | 1.441e-03 | 2.671122e-02 |
| Woma_00012533-RB | FBgn0033061 | SmydA-5, SET and MYND domain containing, arthropod-specific, member 5 | -13332.982019 | -13337.935330 | 9.90662 | 1.647e-03 | 2.905273e-02 |
| Woma_00000675-RA | FBgn0037218 | Gene=aux, Auxilin | -8169.459507 | -8174.393309 | 9.8676 | 1.682e-03 | 2.905273e-02 |
| Woma_00003748-RA | FBgn0037881 | Gene=GCC88, GRIP and coiled-coil domain containing 88 kDa | -15179.276711 | -15184.104795 | 9.65616 | 1.887e-03 | 3.026041e-02 |
| Woma_00012353-RB | FBgn0039528 | Gene=dsd, distracted | -24477.794386 | -24482.606668 | 9.62456 | 1.920e-03 | 3.026041e-02 |
| Woma_00010519-RA | FBgn0052190 | Gene=NUCB1, NUCB1 | -8681.032986 | -8685.830708 | 9.59544 | 1.951e-03 | 3.026041e-02 |
| Woma_00005480-RB | FBgn0038065 | Gene=Snx3, Sorting nexin 3 | -2477.376080 | -2481.989967 | 9.22778 | 2.384e-03 | 3.548615e-02 |
| Woma_00007943-RA | FBgn0037376 | Gene=Hat1, Histone acetyltransferase 1 | -9415.611997 | -9420.223187 | 9.22238 | 2.391e-03 | 3.548615e-02 |
| Woma_00012568-RA | FBgn0050010 | Gene=CG30010 | -4536.353786 | -4540.627293 | 8.54702 | 3.461e-03 | 4.962943e-02 |
